# Impact of Elevated Liver Enzymes on the Severity of Clinical Course of COVID-19: A Retrospective Study From Saudi Arabia

**DOI:** 10.1155/ijh/7385050

**Published:** 2025-11-26

**Authors:** Eyad Makkawy, Shaden Mohammed Alamro, Safiya Ibn Awadh, Reem Basalasil, Ziyad Alkhelaiwi, Bassam AL-Mutairi, Fatimah Rebh

**Affiliations:** ^1^Department of Gastroenterology, Prince Mohammed Bin Abdulaziz Hospital, Riyadh, Riyadh Province, Saudi Arabia; ^2^Department of Internal Medicine, King Abdullah Bin Abdulaziz University Hospital, Riyadh, Riyadh Province, Saudi Arabia; ^3^Department of Gastroenterology, King Faisal Specialist Hospital and Research Centre, Riyadh, Riyadh Province, Saudi Arabia; ^4^Department of Internal Medicine, Prince Mohammed Bin Abdulaziz Hospital, Riyadh, Riyadh Province, Saudi Arabia; ^5^Department of Critical Care Medicine, King Fahad Specialist Hospital, Al-Qassim, Al-Qassim Province, Saudi Arabia; ^6^Department of Intensive Care, Prince Mohammed Bin Abdulaziz Hospital, Riyadh, Riyadh Province, Saudi Arabia; ^7^Department of Adult Infectious Diseases, Prince Mohammed Bin Abdulaziz Hospital, Riyadh, Riyadh Province, Saudi Arabia

**Keywords:** COVID-19, hepatitis enzymes, liver enzymes, mortality, predictor factors, severity

## Abstract

**Background:**

According to recent research, severe acute respiratory syndrome coronavirus 2 (SARS-CoV-2) can cause liver injury, which may be linked to a poorer prognosis. In this retrospective study, we evaluated the incidence of high liver enzyme levels in patients with COVID-19 and their correlation with the severity and prognosis of clinical outcomes.

**Methods:**

A retrospective cohort study was performed from March 2020 to October 2020 at Prince Mohammed Bin Abdulaziz Hospital (PMAH), Riyadh, Saudi Arabia. Demographic information of COVID-19 patients as well as data on clinical features and laboratory parameters were collected. Pearson's correlation (*r*) test was used to assess the correlation between elevated liver enzymes and COVID-19 severity. The multivariate logistic binary regression analysis was used to identify the predictors of elevated liver enzyme levels and mortality among patients with COVID-19.

**Results:**

This cohort included 1033 patients, 73% of whom were male, with a mean age of 49.9 years. Elevated liver enzymes were observed in 52.7% of patients, most commonly with a hepatitis pattern (63.1%). Elevated levels of hemoglobin, creatine kinase–myocardial band, and C-reactive protein, as well as pneumoniae, the requirement of an intensive care unit, comorbidities, and the use of paracetamol, *β*-lactamase, and steroids were significant predictors of elevated liver enzymes (*p* < 0.05). Interestingly, Saudi patients (*p* = 0.019) were found to be a significant protective predictor of elevated liver enzymes. Our findings revealed that elevated liver enzyme levels were significantly correlated with the severity of COVID-19 (*p* < 0.05) in terms of qSOFA score. Moreover, older age, diabetes, qSOFA score, and elevated hepatitis enzymes were associated with mortality (*p* = 0.043).

**Conclusions:**

Elevated liver enzyme levels were common in patients with COVID-19 and were associated with the severity and prognosis of clinical outcomes.

## 1. Introduction

By late 2019, a highly contagious novel coronavirus broke out in the Chinese province of Wuhan infecting millions of people worldwide [1]. On March 12, 2020, the severe acute respiratory syndrome coronavirus 2 (SARS-CoV-2) or coronavirus disease 2019 (COVID-19) was declared a pandemic by the World Health Organization (WHO). On May 7, 2020, more than 3.84 million cases of COVID-19 had been reported in over 187 countries and territories, resulting in more than 269,000 deaths [2].

Patients diagnosed with COVID-19 are either asymptomatic, or manifesting the general signs and symptoms of flu including fever, shortness of breath, cough, and oxygen desaturation [3, 4]. The main viral receptor of COVID-19 is the angiotensin-converting enzyme 2 (ACE2), which is distributed in most of the body organs including the liver, the pancreas, the kidneys, and the heart, which allows COVID-19 to cause a systemic infection [5].

The incidence of higher levels of liver enzymes in patients admitted with COVID-19 ranges from 14% to 53%, primarily in a hepatocellular pattern (elevated aspartate aminotransferase [AST] and alanine transaminase [ALT] and slightly elevated bilirubin) [6, 7]. COVID-19-associated liver injury is defined as any liver damage occurring during disease progression and treatment of COVID-19 in patients with or without preexisting liver diseases [8].

A recent study done by Guan et al., which included 1099 COVID-19 cases from China, demonstrated that elevated ALT and AST levels occurred in 21.3% and 22.2% of cases, respectively. Furthermore, a higher level of ALT was detected in severe cases, compared with nonsevere ones (28.1% vs. 19.8%), as well as a higher level of AST (39.4% vs. 18.2%) [9].

Data about the impact of elevated liver enzyme levels on the severity and prognosis of clinical outcomes of COVID-19 is limited. Hence, the aims of this retrospective study were to estimate the incidence of elevated liver enzyme levels among COVID-19 patients and investigate their correlation with the severity and prognosis of clinical outcomes, which will give a better approach to managing patients with COVID-19.

## 2. Methods

### 2.1. Study Design

This study is a retrospective observational study of liver enzyme levels in patients admitted with COVID-19 infection and its correlation with the severity and prognosis of the disease.

The data was collected from March 2020 to October 2020. Patients admitted to the hospital as a case of COVID-19 were included in this study if they met the following criteria: (1) confirmed SARS-CoV-2 by RT-PCR, (2) adults ≥ 14 years old, (3) patients admitted to the regular ward or intensive care unit (ICU), and (4) patients confirmed with COVID-19 with elevated liver enzymes defined as a rise in ALT, AST, alkaline phosphatase (ALP), gamma-glutamyl transferase (GGT), or total bilirubin and must have at least two abnormal readings of elevated liver function throughout their admission. Patients with preexisting liver disease were excluded if identified through documented history, abnormal baseline liver tests, radiological evidence of chronic liver disease, or histopathological/endoscopic reports.

### 2.2. Ethical Approval

The data were collected and registered through the person assigned by the principal investigator. Data were entered in an Excel sheet as per the follow-up assessment's tool mentioned above. Only the investigator had access to the Excel sheet (for data entry, follow-up, and rechecking the data). The data were anonymous and protected. Investigators guaranteed that clinical data would be used for clinical research only.

### 2.3. Data Collection

Relevant information of all enrolled patients was retrieved from the hospital's electronic HIS (Cerner) system, including the following data: (1) baseline demographic data (gender, body mass index [BMI], age, and nationality), (2) chronic medical illness (e.g., diabetes mellitus, hypertension, and chronic kidney disease), (3) clinical symptoms (e.g., respiratory symptoms, gastrointestinal symptoms, and cardiovascular symptoms), (4) presence of pneumoniae, (5) hospital length of stay, (6) need for ICU admission and length of stay in ICU, (7) development of medical complications, (8) the severity of COVID-19 infection measured by the quick Sequential Organ Failure Assessment (qSOFA) score, and (9) laboratory result for ALT, AST, GGT, ALP, and total bilirubin in addition to other laboratory data requested during the patient's hospital stay (e.g., white blood cell, blood platelet, hemoglobin, ferritin, and creatinine).

Elevated liver enzymes are classified into a hepatitis pattern or a cholestatic pattern. The hepatitis pattern was defined as a predominant elevation of ALT and/or AST (≥ 3× the upper limit of normal), whereas the cholestatic pattern was defined as a disproportionate elevation in ALP and/or GGT (≥ 2× the upper limit of normal), with or without hyperbilirubinemia [10].

### 2.4. Outcomes

The primary outcome was the incidence of elevated liver enzymes in patients with COVID-19 and the association of elevated liver enzyme levels with the severity and prognosis of COVID-19 infection. The secondary outcome was the evaluation of any confounding factors that may predict the elevation of liver enzymes (patients' characteristics, clinical outcomes, laboratory measures, and use of medications).

### 2.5. Statistical Analysis

The collected data were analyzed using the Statistical Package for the Social Sciences (SPSS) for Windows. The mean and standard deviation (SD) were used to describe the continuous variables, while the frequency and percentages were applied to describe categorical measures. Pearson's correlation (*r*) test was used to assess the correlation between elevated liver enzymes levels and COVID-19 severity. The multivariate logistic binary regression analysis was used to identify the predictive factors of elevated liver enzymes levels and mortality among patients with COVID-19 by calculating the odds ratio (OR) with a 95% confidence interval (CI). *p* values of less than 0.05 were considered for statistical significance.

## 3. Results

### 3.1. Demographic and Clinical Characteristics of the Patient Cohort

Our study included 1033 COVID-19 patients, predominantly male (73.4%), with a mean (SD) age of 49.92 (15.23) years. The age group 51–60 years constituted 23.4% of the cohort followed by 31–40 years (22.3%). The majority of patients were non-Saudi (59.8%). The mean BMI was 28.87 (6.20), and overweight patients made up 40.3%. Then, 55.2% of patients had comorbidities, mainly diabetes mellitus (62.5%) and hypertension (52.3%). Among 866 patients, common symptoms included unrecorded fever (57.6%), malaise (20.1%), and fever below 39°C (23%). Respiratory symptoms were reported by 894 patients, predominantly shortness of breath (75.7%), pneumoniae (69%), and dry productive cough (46.2%).

Gastrointestinal symptoms were noted in 291 patients, mainly diarrhea (46.7%) and vomiting (32.3%). Cardiovascular symptoms (*n* = 45) included chest pain or pleuritic chest pain (84.4%). Regarding medication use after admission, paracetamol was administered to 82.1%, and beta-lactamase antibiotics were administered to 83.1%. Hydroxychloroquine was given to 10.5%, statins to 17.8%, and steroids to 58.1%.

The mean duration of symptoms was 6.20 (3.76) days. The average hospital stay was 13.11 (16.68) days, with 61.3% of patients in required supplemental O_2_ and 23.2% in required ICU admission. The average ICU length of stay was 19.16 (9) days. Endotracheal intubation was required by 14% of patients. Medical complications occurred in 19% of patients, mainly respiratory failure (76.5%). Regarding the severity of the clinical course of COVID-19, the overall mean qSOFA score was 0.10 (0.30) points for all the patients upon admission. However, it was 0.30 (0.50) points for the ICU admitted patients. The survival rate was 94.5%. Among patients who died (*n* = 57), the majority had a hepatitis pattern (*n* = 49), while only eight patients had a normal pattern of raised liver enzymes (Table 1 and Table S1).

### 3.2. Analysis of Laboratory Findings Including LFT Results at Admission

The mean white blood cell, blood platelet, and absolute lymphocyte count, were 7.51 × 10^9^/L (4.03), 233.63 × 10^9^/L (88.72), and 4.97 × 10^9^/L (1.66), respectively. Serum hemoglobin averaged 13.73 g/L (1.98), and 37.9% of patients had low hemoglobin levels. The majority of patients had elevated levels of ferritin (54.2%) and normal levels of fibrinogen (61.3%). Similarly, the majority of patients had elevated levels of creatine kinase–myocardial band (CK-MB) (53.1%) and C-reactive protein (CRP) (58.7%).

Liver function tests at admission were predominantly normal. The mean ALP level was 77.92 U/L (47.46), AST was 65.54 U/L (190.17), and ALT was 49.22 U/L (56.93). The mean GGT level was 108.1 U/L (112.94), and total bilirubin was 11.39 *μ*mol/L (7.49). Elevated liver enzymes were observed in 52.7% of patients, with a hepatitis pattern in 63.1% and a cholestatic pattern in 1.3% (Table 2).

### 3.3. Predictive Factors of Elevated Liver Enzymes

Multivariate binary logistic regression analysis revealed that Saudi patients were found to be significantly less predicted (OR = 0.658, 95% CI: 0.464–0.934) to present with elevated liver enzymes compared to expatriate patients (*p* = 0.019). However, elevated levels of hemoglobin (*p* = 0.012), CK-MB (*p* = 0.001), and CRP (*p* = 0.016) were significant predictors of elevated liver enzymes. Similarly, patients presenting with pneumoniae (*p* = 0.038) and comorbidities (*p* = 0.011) and who required admission to the ICU immediately upon admission (*p* = 0.001) were found to be significant predictors of elevated liver enzymes. Regarding medication use after admission, paracetamol (*p* < 0.001), *β*-lactamase (*p* < 0.001), and steroid (*p* = 0.026) were significant predictors of elevated liver enzymes (Table 3). The remaining parameters listed in Tables 1 and 2 were found not significant.

### 3.4. Correlations Between Liver Enzyme Elevations and Severity of Disease

Pearson's correlations (*r*) test identified a significant positive correlation between elevated levels of ALT and AST (*r* = 0.507, *p* < 0.01) and between elevated levels of ALT and total bilirubin (*r* = 0.461, *p* < 0.01) and AST and total bilirubin (*r* = 0.339, *p* < 0.01). However, elevated levels of ALP showed no significant correlation with elevated levels of ALT and AST but were positively correlated with elevated levels of GGT (*r* = 0.364, *p* < 0.01). Also, elevated levels of all liver enzymes correlated significantly and positively with qSOFA score (*p* < 0.05 or *p* < 0.01), indicating a significant association between elevated liver enzymes and COVID-19 severity (Table 4).

### 3.5. Predictive Factors of Mortality in COVID-19 Patients

Multivariate analysis of predictive factors of mortality in COVID-19 patients demonstrated that older age (OR = 1.075, *p* < 0.001), diabetes (OR = 2.596, *p* = 0.006), and qSOFA score (OR = 11.457, *p* < 0.001) were strong predictors of mortality. Furthermore, elevated hepatitis enzymes significantly increased mortality (OR = 1.208, *p* = 0.043), whereas elevation of cholestatic enzymes did not (OR = 0.815, *p* = 0.292). Neither sex nor BMI was associated with increased risk of mortality in COVID-19 patients (Table 5).

## 4. Discussion

Our study including 1033 COVID-19 patients admitted at Prince Mohammed Bin Abdulaziz Hospital (PMAH) provides insights into the correlation between elevated liver enzymes and the severity of clinical outcomes and mortality among COVID-19 patients. These findings contribute to the broader understanding of COVID-19's multiorgan impact, specifically its ability to disrupt liver function.

A notable observation was the high prevalence of elevated liver enzymes (52.7%), predominantly exhibiting a hepatitis pattern (63.1%). This reinforces the notion that COVID-19 can inflict directly or indirectly liver injury, potentially exacerbating preexisting liver conditions. In the same context, a meta-analysis demonstrated that Chinese patients with severe or critical illness from COVID-19 are more likely to manifest with abnormal liver chemistries [11].

We observed a relatively low prevalence of cholestasis (1.3%), suggesting that cholestatic injury remains a rare entity within COVID-19, similar to existing literature [12]. Interestingly, Saudi nationality emerged as a protective factor against elevated liver enzymes compared to non-Saudi patients. The finding that Saudi nationality was associated with a lower likelihood of elevated liver enzymes is intriguing. Several possible explanations may contribute to this observation. Differences in demographic profiles—such as age distribution, prevalence of comorbidities, or BMI—could partly explain this association. In addition, health system–related factors, including earlier access to care, better follow-up, and timely intervention among Saudi patients, may play a role. It is also conceivable that genetic or lifestyle factors could underlie this pattern [13]. However, it is important to emphasize that, given the retrospective design of our study, no causal inference can be made, and these associations should be interpreted cautiously.

Picon et al. also reported that upon admission, abnormal liver enzymes were found in more than half of their patients (58.6%) [14]; this percentage falls within a broad range (14%–81.7%) reported by other studies that also found abnormal liver enzymes during hospitalization or upon admission [6, 15–17]. This variety is attributed to the major differences across the examined populations as well as the various liver enzyme cutoffs that were applied.

Our analysis also identified specific biomarkers, such as elevated hemoglobin, CK-MB, and CRP levels, as significant risk factors for liver enzyme elevations. This aligns with recent studies indicating a potential link between systemic inflammation, cellular injury, and hepatic dysfunction in COVID-19 patients. The significance of these biomarkers underscores the need for comprehensive monitoring in COVID-19 patients, including liver function tests and inflammatory markers [15, 18, 19]. Zhu et al. also reported that the severity of COVID-19 can be predicted by inflammatory indicators like CRP [20].

The potential confounding effect of medication use upon admission is an important consideration in our analysis. We observed higher odds of elevated liver enzymes in patients who had been administered medications such as paracetamol, beta-lactamase antibiotics, and steroids. An important consideration is that patients receiving medications such as paracetamol, beta-lactam antibiotics, and steroids may have represented a subgroup with more severe illness, necessitating more aggressive treatment. This greater baseline severity could partly explain the observed association between these drugs and elevated liver enzyme levels. While drug-induced hepatotoxicity is a plausible contributor, our data cannot fully distinguish between medication effects and the impact of disease severity as confounding factors. It suggests that while these medications might contribute to liver enzyme elevation, they are unlikely to be the sole cause. A key limitation of our study is the inability to ascertain the exact dosage and frequency of these medications for each patient. Additionally, the lack of baseline liver enzyme levels prior to medication administration limits our ability to fully understand the impact of these drugs on liver function. This gap in data poses a challenge in disentangling the direct effects of COVID-19 from those potentially induced by medication. Savransky et al. concluded that for the purpose of treating COVID-19 in these patients, nonhepatotoxic medications should be taken into consideration. It is unclear if COVID-19 patients with chronic liver disease would have a different course of disease or die from it. Moreover, liver injury may result from hypoxia brought on by significant COVID-19 brought on by acute respiratory distress syndrome [21].

New drugs are always being developed, particularly for COVID-19. This means that there are always new therapy options that may be more harmful and hepatotoxic, have fewer side effects, or possibly do both. The majority of drugs, in fact, have unfavorable side effects. The majority of the time, drug-induced liver damage is not severe, and the prescribing doctor should be able to carefully weigh the advantages of the medications against any possible drawbacks. To prevent worsening circumstances, it is necessary to be able to identify and control the sporadic negative impacts [22].

Our findings also align with existing literature showing a significant association between elevated liver enzymes and severe disease manifestations [15, 18, 19]. We found that severe manifestations like pneumonia, comorbidities, and ICU admission were associated with higher odds of elevated liver enzymes, which are in line with the current literature [23]. COVID-19 primarily affects the lungs, where it progresses through the ACE2 receptor to cause pneumonia. The infection can spread, via the ACE2 receptors, to a number of organs, including the brain, heart, liver, kidney, gastrointestinal tract, endothelium, immune system, and red blood cells, depending on the viral load. Cytokine storm, an unregulated immune system's widespread release of proinflammatory cytokines, may accelerate this process [24]. This complex relationship necessitates a nuanced approach to managing COVID-19 patients, especially those with preexisting medical conditions.

Various mechanisms may account for the frequency and severity of liver involvement in COVID-19. Our study, however, does not thoroughly investigate the mechanisms behind these correlations. The widespread presence of the ACE2 receptor in human tissues may be a potential factor in direct cellular damage [25]. Furthermore, severe hypoxic respiratory failure can contribute to a component of hypoxic hepatitis in patients [26]. Other factors contributing to liver injury include hepatic vascular thrombosis, extensive systemic inflammation, and drug-related toxicity [27, 28].

This study found a significant positive correlation between ALT and AST levels and between both enzymes' levels and total bilirubin level. In a similar vein, Phipps et al. discovered that assessed clinical outcomes and overall disease severity were substantially correlated with higher ALT values. Elevated AST and ALT levels are often observed in COVID-19 patients in the United States, occurring in 38%–63% and 29%–39% of cases, respectively [29]. Picon et al. also showed that mild AST and/or ALT abnormalities are predictive of the severe clinical course of COVID-19, and the prevalence of aberrant AST and/or ALT at admission is more than 50% [14].

Our study revealed that older age, diabetes, qSOFA score, and hepatitis enzymes are strong predictors of mortality. A previous observational case-control study supported this finding linking COVID-19 to poorer outcomes and increased mortality, including raised D-dimer concentration and older age [30]. The presence of hepatitis pattern enzyme elevations adds incremental prognostic information, while qSOFA remains a strong predictor of mortality. This highlights the potential clinical value of incorporating liver function tests alongside qSOFA in risk stratification. Unlike the hepatitis pattern, which we found to be a predictor of increased mortality risk, cholestatic injury appeared to be a less critical prognostic factor. This disparity suggests different underlying mechanisms of liver injury in COVID-19, which could be explored in future studies to better understand these pathophysiological processes.

Our study has limitations, including its single-center design and lack of long-term follow-up data, which may impact the generalizability and comprehensiveness of our findings. Additionally, our inability to track liver enzyme trends or assess the impact of antiviral medications throughout a patient's hospital stay represents a gap in our analysis. Finally, the retrospective design, the presence of missing or incomplete data, and the restriction of follow-up to in-hospital outcomes represent important limitations that should be taken into account when interpreting the findings of this study.

## 5. Conclusions

Our study offers valuable insights into the factors influencing liver injury in COVID-19 patients. It underscores the importance of careful monitoring of liver function and medication regimens in these patients. Future research should focus on elucidating the mechanisms underlying different patterns of liver injury in COVID-19, enhancing our understanding and management of hepatic complications in this patient population.

## Figures and Tables

**Table 1:** Demographic and clinical characteristics of the COVID-19 patients (*n* = 1033).

**Parameter**	**N** **(%) or mean (SD)**
Sex, *n* (%)	
Female	275 (26.6)
Male	758 (73.4)
Age (years), mean (SD)	49.92 (15.23)
Age group, *n* (%)	
≤ 30 years	90 (8.7)
31–40 year	230 (22.3)
41–50 years	221 (21.4)
51–60 years	242 (23.4)
61–70 years	150 (14.5)
≥ 71 years	100 (9.7)
Nationality, *n* (%)	
Non-Saudi	618 (59.8)
Saudi	415 (40.2)
Body mass index (BMI) score, mean (SD)	28.87 (6.20)
Body mass index level, *n* (%)	
Underweight	30 (2.9)
Normal	201 (19.5)
Overweight	416 (40.3)
Obese Type I	227 (22)
Obese Type II	159 (15.4)
Comorbidity, *n* (%)	
No	463 (44.8)
Yes	570 (55.2)
Duration of symptoms in days, mean (SD)	6.20 (3.76)
Hospital length of stay days, mean (SD)	13.11 (16.68)
Required supplemental O_2_, *n* (%)	633 (61.3)
Required ICU admission, *n* (%)	240 (23.2)
ICU length of stay (LOS) days, mean (SD)	19.16 (9)
Required endotracheal intubation (ETT), *n* (%)	145 (14)
Developed medical complications, *n* (%)	196 (19)
qSOFA, mean (SD)	0.10 (0.30)
qSOFA for the ICU admitted patients, mean (SD)	0.30 (0.50)
Final hospital disposition, *n* (%)	
Survived	976 (94.5)
Died	57 (5.5)

**Table 2:** Descriptive analysis of laboratory results and liver function tests at admission among COVID-19 patients.

**Parameter**	**N** **(%) or mean (SD)**
White blood cell count ×10^9^/L, mean (SD)	7.51 (4.03)
Blood platelet ×10^9^/L, mean (SD)	233.63 (88.72)
Absolute lymphocytes ×10^9^/L, mean (SD)	4.97 (1.66)
Serum hemoglobin g/dL, mean (SD)	13.73 (1.98)
Serum hemoglobin g/dL level, *n* (%)	
Low < 12 g/dL	392 (37.9)
Normal 12–18 g/dL	635 (61.5)
Elevated > 18 g/dL	6 (0.6)
Ferritin level ng/mL, *n* (%)	
Low < 13 ng/mL	7 (0.7)
Normal 13–400 ng/mL	466 (45.1)
Elevated > 400 ng/mL	560 (54.2)
Fibrinogen level g/L, *n* (%)	
Normal 2–4 g/L	633 (61.3)
Elevated > 4 g/L	400 (38.7)
Serum creatinine *μ*mol/day, mean (SD)	191.84 ± 129.75
CK-MB level U/L, *n* (%)	
Normal < 25 U/L, *n* (%)	484 (46.9)
Elevated ≥ 25 U/L, *n* (%)	549 (53.1)
CRP level mg/L, *n* (%)	
Normal < 3 mg/L	427 (41.3)
Elevated ≥ 3 mg/L	606 (58.7)
Liver function tests	
ALP U/L, mean (SD)	77.92 (47.46)
ALP U/L level, *n* (%)	
Normal 40–150 U/L	1027 (99.4)
Mild elevation > 150–450 U/L	6 (0.6)
AST U/L, mean (SD)	65.54 (190.17)
AST U/L level, *n* (%)	
Normal 5–34 U/L	618 (59.8)
Mild elevation > 34–102 U/L	344 (33.3)
Moderate elevation > 102–204 U/L	60 (5.8)
Severe elevation > 204 U/L	6 (0.6)
Critical elevation > 374 U/L	5 (0.5)
ALT U/L, mean (SD)	49.22 (56.93)
ALT U/L level, *n* (%)	
Normal 5–55 U/L	760 (73.6)
Mild elevation > 55–165 U/L	241 (23.3)
Moderate elevation > 165–330 U/L	29 (2.8)
Severe elevation > 330 U/L	1 (0.1)
Critical elevation > 605 U/L	2 (0.2)
GGT U/L, mean (SD)	108.1 (112.94)
GGT U/L level, *n* (%)	
Normal 12–64 U/L	982 (95.1)
Mild elevation > 64–192 U/L	30 (2.9)
Moderate elevation > 192–384 U/L	16 (1.5)
Severe elevation > 384 U/L	5 (0.5)
Total bilirubin *μ*mol/L, mean (SD)	11.39 (7.49)
Total bilirubin *μ*mol/L level, *n* (%)	
Normal 3.4–20.5 *μ*mol/L	953 (92.3)
Mild elevation > 20.5–61.5 *μ*mol/L	78 (7.6)
Moderate elevation > 61.5–123 *μ*mol/L	2 (0.2)
Elevated of any liver enzymes (out of the five), *n* (%)	
No	489 (47.3)
Yes	544 (52.7)
Number of elevated liver enzymes (ALT, AST, ALP, GGT, and bilirubin), *n* (%)	
None	489 (47.3)
One enzyme	313 (30.3)
Two enzymes	184 (17.8)
Three enzymes	44 (4.3)
Four enzymes	3 (0.3)
Pattern of raised liver enzymes at admission time, *n* (%)	
Normal	282 (27.3)
Mixed	76 (7.4)
Isolated high bilirubin	10 (1)
Hepatitis (ALT/AST)	652 (63.1)
Cholestatic (GGT/ALP)	13 (1.3)

**Table 3:** Multivariate binary logistic regression analysis of predictors of elevated liver enzymes.

**Parameter**	**Multivariate adjusted OR**	**95% CI for OR**		**p** **value**
		**Lower**	**Upper**	
Age (> 60)	0.996	0.983	1.009	0.565
Sex = male	1.279	0.833	1.962	0.260
Body mass index score	1.001	0.974	1.028	0.962
Nationality = Saudi	0.658	0.464	0.934	0.019⁣^∗^
qSOFA severity score	1.203	0.649	2.229	0.558
Hemoglobin level (> 18 g/L)	1.614	1.113	2.340	0.012⁣^∗^
Firbrinogen level (> 4 g/L)	1.424	0.961	2.110	0.078
Elevated serum creatinine (> 110 *μ*mol/L)	0.740	0.506	1.081	0.120
CK-MB level (≥ 25 U/L)	1.790	1.254	2.557	0.001⁣^∗^
C-reactive protein (≥ 3 mg/L)	1.543	1.084	2.197	0.016⁣^∗^
Serum ferritin (> 400 ng/mL)	1.301	0.923	1.833	0.133
Presence of pneumonia	1.642	1.422	1.976	0.038⁣^∗^
Requirement of ICU	1.399	1.232	1.684	0.001⁣^∗^
Comorbidity	1.606	1.412	1.890	0.011⁣^∗^
Paracetamol	1.587	1.360	1.852	< 0.001⁣^∗^
*β*-Lactamase	1.932	1.588	2.351	< 0.001⁣^∗^
Steroid	1.195	1.022	1.398	0.026⁣^∗^

*Note:* Dependent variable = had admission time rise in one or more liver enzymes (no/yes).

⁣^∗^Significant *p* value.

**Table 4:** Correlations between elevated liver enzymes and COVID-19 severity.

**Parameter**	**ALT**	**AST**	**ALP**	**GGT**	**Bilirubin**
ALT	1				
AST	0.507⁣^∗^				
ALP	−0.011	0.003			
GGT	0.060	0.005	0.364⁣^∗^		
Total bilirubin	0.461⁣^∗^	0.339⁣^∗^	0.013	−0.006	
qSOFA score	0.111⁣^∗^	0.136⁣^∗^	0.072⁣^∗^	0.075⁣^∗^	0.077⁣^∗^

⁣^∗^*p* value < 0.050.

**Table 5:** Multivariate binary logistic regression analysis of predictors of mortality.

**Parameter**	**Multivariate adjusted odds ratio**	**95% CI for OR**		**p** **value**
		**Lower**	**Upper**	
Sex = male	0.905	0.451	1.819	0.780
Age (> 60)	1.075	1.049	1.102	< 0.001⁣^∗^
Body mass index	0.974	0.919	1.033	0.378
Diabetic	2.596	1.307	5.156	0.006⁣^∗^
qSOFA score	11.457	5.679	23.116	< 0.001⁣^∗^
Hepatitis enzymes (ALT/AST)	1.208	1.006	1.451	0.043⁣^∗^
Cholestatic enzymes (GGT/ALP)	0.815	0.558	1.192	0.292

⁣^∗^Significant value.

## Data Availability

The data that support the findings of this study are available from the corresponding author upon reasonable request.

## Supporting Information

Supporting Information Additional supporting information can be found online in the Supporting Information section. Table S1 reported data about the type of comorbidity, symptoms, medication use after admission, and medical complications of the COVID-19 patients.
