# Supplementary material for: Impact of Elevated Liver Enzymes on the Severity of Clinical Course of COVID-19: A Retrospective Study From Saudi Arabia
Source: Int J Hepatol. 2025 Nov 26;2025:7385050. doi: 10.1155/ijh/7385050 (PMC12674859; doi:10.1155/ijh/7385050)
Supplement: Supporting Information — Additional supporting information can be found online in the Supporting Information section. Table S1 reported data about the type of comorbidity, symptoms, medication use after admission, and medical complications of the COVID-19 patients. [file 7385050.f1.doc]

**Supplementary table 1.** Type of comorbidity, symptoms, medication uses after admission, and medical complications of the COVID-19 patients (n=1033)

| **Parameter** | | | | | **N (%) or mean (SD)** | | |
| --- | --- | --- | --- | --- | --- | --- | --- |
|  | **Type of Comorbidity, n (%)** | | | | |  | |
|  | Diabetes mellitus | | | 356 (62.5) | |  | |
|  | Hypertension | | | 298 (52.3) | |  | |
|  | Chronic Kidney Disease | | | 17 (3) | |  | |
|  | End-stage renal disease (ESRD) | | | 16 (2.8) | |  | |
|  | Malignancy | | | 13 (2.3) | |  | |
|  | Autoimmune Disease | | | 9 (1.6) | |  | |
|  | COPD and /or Asthma | | | 74 (13) | |  | |
|  | Dyslipidaemia | | | 20 (3.5) | |  | |
|  | Thyroid Disorder | | | 30 (5.3) | |  | |
|  | Central Nervous System Disorder | | | 22 (3.9) | |  | |
|  | Cardiovascular syndrome (IHD, RHD, HF) | | | 80 (14) | |  | |
|  | **General symptoms, n=866, n (%)** | | | | | |  |
|  | Headaches | 83 (9.6) | | | | |  |
|  | Myalgia and Body Aches | 5 (0.6) | | | | |  |
|  | Loss of consciousness/confusion | 4 (0.5) | | | | |  |
|  | Loss of smell and / taste | 2 (0.2) | | | | |  |
|  | Malaise | 174 (20.1) | | | | |  |
|  | Undocumented fever | 499 (57.6) | | | | |  |
|  | Fever > 37.3 < 39 Degrees Celsius | 199 (23) | | | | |  |
|  | Fever ≥ 39 Degrees Celsius | 132 (15.2) | | | | |  |
|  | Other General Symptoms | 5 (0.6) | | | | |  |
|  | **Respiratory Symptoms, n=894, n (%)**  Pneumoniae  713 (69) | | | | | |  |
|  | Runny nose | 9 (1) | | | | |  |
|  | Pleuritic chest pain | 21 (2.3) | | | | |  |
|  | Productive cough | 248 (27.7) | | | | |  |
|  | Dry Productive cough | 413 (46.2) | | | | |  |
|  | Sore throat | 84 (9.4) | | | | |  |
|  | Shortness of breathing | 677 (75.7) | | | | |  |
|  | **Gastrointestinal symptoms, n=291, n (%)** | | | | | |  |
|  | Anosmia | 2 (0.7) | | | | |  |
|  | Constipation | 3 (1) | | | | |  |
|  | Anorexia | 83 (28.5) | | | | |  |
|  | Diarrhea | 136 (46.7) | | | | |  |
|  | Abdominal Pain | 34 (11.7) | | | | |  |
|  | Nausea | 74 (25.4) | | | | |  |
|  | Vomiting | 94 (32.3) | | | | |  |
|  | Other GI complaints | 1 (0.3) | | | | |  |
|  | **Cardiovascular symptoms, n=45, n (%)** | | | | | |  |
|  | Chest Pain/ Pleuritic chest pain | 38 (84.4) | | | | |  |
|  | Dizziness | 7 (15.6) | | | | |  |
|  | Sweating | 9 (20) | | | | |  |
|  | **Medication uses after admission**  Paracetamol | 835 (82.1) | | | | |  |
|  | Beta-lactamase antibiotics | 845 (83.1) | | | | |  |
|  | Hydroxychloroquine | 107 (10.5) | | | | |  |
|  | Statins | 181 (17.8) | | | | |  |
|  | Steroids | 591 (58.1) | | | | |  |
| **Medical complications, n=196, n (%)** | | | | | | | |
| Multi-organ failure | | | 5 (2.6) | | | | |
| Central Line Central-related bloodstream infection | | | 7 (3.6) | | | | |
| Respiratory Failure | | | 150 (76.5) | | | | |
| Thromboembolic event | | | 15 (7.7) | | | | |
| Septic Shock | | | 20 (10.2) | | | | |
| Stroke | | | 3 (1.5) | | | | |
| Acute Coronary Syndrome/cardiac complications | | | 45 (23) | | | | |
| Acute Respiratory Distress Syndrome | | | 16 (8.2) | | | | |
| Acute Kidney Injury | | | 8 (4.1) | | | | |
